# Variations of risk factors for ischemic stroke and its subtypes in Chinese patients in Taiwan

**DOI:** 10.1038/s41598-021-89228-x

**Published:** 2021-05-06

**Authors:** Chung-Fen Tsai, Cathie L. M. Sudlow, Niall Anderson, Jiann-Shing Jeng

**Affiliations:** 1 Department of Neurology, Cardinal Tien Hospital, Fu Jen Catholic University, New Taipei City, Taiwan; 2 Centre for Clinical Brain Sciences, University of Edinburgh, Edinburgh, UK; 3 Centre for Population Health Sciences, University of Edinburgh, Edinburgh, UK; 4 Stroke Center and Department of Neurology, National Taiwan University Hospital, No 7, Chung-Shan South Road, Taipei, 100 Taiwan

**Keywords:** Neurology, Risk factors

## Abstract

Chinese have a higher stroke incidence and a different distribution of ischemic stroke (IS) subtypes as compared with Caucasians. Herein we aimed to investigate the prevalence and associations of major risk factors in IS and its subtypes in Chinese patients. From 2006 to 2011, we included 4953 acute IS patients consecutively recruited in National Taiwan University Hospital Stroke Registry (mean age 68 years; male 59%). For each risk factor, we accessed the proportion in all IS patients, and calculated odds ratios for each main IS subtype versus other subtypes. Multiple logistic regression models were used to adjust for confounders, and to examine the associations of risk factors with IS subtypes. Compared with other ischemic subtypes, large artery atherosclerotic and lacunar strokes were associated with hypertension, diabetes, and hyperlipidaemia, while cardioembolic strokes were associated with ischemic heart disease. Furthermore, the associations with hypertension and diabetes became stronger in lacunar strokes after adjusting for confounders, but not in other ischemic subtypes. Here we report the variable effects of risk factors on different IS subtypes in Chinese patients in Taiwan. Our findings could help shed light on different mechanisms of IS subtypes and provide targets to make more effective strategies for IS prevention.

## Introduction

Although incidence and mortality of stroke tend to decrease in recent 20 years, the global stroke burden continues to increase, especially in developing countries^1^. Compared with western populations, Chinese have a higher incidence and mortality of stroke, along with high disability-adjusted life-years^2^. The stroke burden on Chinese populations is quite heavy, particularly from ischemic stroke (IS)^3,4^.

Our previous systematic review has demonstrated a younger onset, and a different distribution of IS subtypes, especially a higher proportion of lacunar infarct (LI) in Chinese versus Caucasian patients^5^. Yet questions such as what cause the differences remain unanswered. They may relate to differences in genetics, prevalence and associations of risk factors with IS subtypes between populations^6–8^. Further meta-analyses have shown that Chinese IS patients have similar prevalence of hypertension, diabetes, smoking and alcohol, but lower prevalence of atrial fibrillation (AF), ischemic heart disease (IHD) and hypercholesterolemia as compared with Caucasian patients, while associations of risk factors with IS subtypes were similar^9^. However, these findings could be confounded by age, sex, and other risk factors. In addition, they may be driven by risk factor dependent nature of the classification system.

To overcome the confounding effects, and to test the hypothesis that risk factors varied among IS subtypes, we conducted individual patient analyses using data from the National Taiwan University Hospital (NTUH) Stroke Registry. We aimed to evaluate the risk factors distribution in all IS and subtypes, to assess the associations with each main IS subtype versus others using both risk-factor dependent and risk-factor free classification schemes, and to adjust for confounders.

## Methods

### Subjects

We included 4953 acute IS Chinese patients from the NTUH Stroke Registry, which had prospectively recruited consecutive acute stroke patients with onset in 10 days. The mean age was 68.1 (± 13.8) years; 59% were men. Informed consent was obtained from all participants or their families^10^. Neurologists assessed the patients, collected medical history and clinical information, and arranged timely brain computed tomography (CT)/magnetic resonance imaging (MRI). The stroke registry and this study protocol had been approved by the NTUH and Cardinal Tien Hospital Institutional Review Boards, and all research was performed in accordance with relevant guidelines/regulations. Herein, we analysed acute IS adult patients from January 2006 to December 2011.

### Diagnosis of ischemic stroke and its subtypes

The diagnosis of IS was based on the definition from World Health Organization, and confirmed by brain CT/MRI^11^. IS patients were classified into five etiological subtypes according to the TOAST criteria (risk-factor dependent)—large artery atherosclerosis (LAA: infarct lesions greater than 1.5 cm on CT/MRI with more than 50% stenosis of an intracranial or extracranial artery by duplex imaging or CT angiography (CTA)/MR angiography (MRA)/angiography, and excluding cardiogenic embolism), LI (lacunar syndrome with infarct lesion less than 1.5 cm on brain CT/MRI, and excluding cardiac embolism and large artery stenosis more than 50% ), cardioembolism (CE: at least one cardiac source of emboli such as atrial fibrillation being identified, and excluding large artery atherosclerosis), other determined, and undetermined etiology. We also classified these IS patients into four anatomical subtypes of the Oxfordshire Community Stroke Project (OCSP classification)—LI, total and partial anterior circulation infarct (TACI and PACI), and posterior circulation infarct (POCI)^12,13^. The OCSP classification system is a risk-factor free scheme based on clinical features of stroke syndrome and brain image findings, being free of etiological assumptions of risk factors to reduce the possibility of classification bias. Patients with previous stroke and its complications, intracerebral/subarachnoid/subdural/epidural haemorrhage, tumor, non-cerebrovascular causes or no brain imaging were excluded.

### Major risk factor definitions

Major risk factors were defined as follows: hypertension (history, antihypertensive treatment or blood pressure ≥ 140/90 mmHg 7 days after stroke), diabetes (history, anti-diabetic treatment, or fasting plasma glucose ≥ 126 mg/dl), AF (history or electrocardiographic evidence), IHD (history or electrocardiographic evidence), hyperlipidemia (history, anti-hyperlipidemic medication, hypercholesterolemia or hypertriglyceridemia ≥ 200 mg/dl), current or previous smoking, and alcohol intake (habitual drinking more than once per week). We also recorded previous stroke and history of transient ischemic attack (TIA).

### Statistical analysis

We used analysis of variance (ANOVA) to compare mean age, and chi-square test to compare proportions of male sex and risk factors among IS subtypes. We divided age into five equal-sized categories as it was not assumed to contribute a linear change, and calculated proportions of risk factors in each IS subtype. For each risk factor, we computed crude odds ratio (OR) with 95% confidence interval (CI) for LAA, LI, and CE versus other subtypes in the TOAST classification, and used logistic regression to adjust for age and sex and to examine if there was any interaction, obtaining adjusted ORs (OR I). Then we developed a second logistic regression model to adjust for age, sex, and all major risk factors, and used stepwise selection to incorporate the significant 2-way interactions among variables (*p* < 0.001), obtaining adjusted ORs (OR II). Also, we categorized OCSP subtypes into LI and non-lacunar infarct (non-LI), doing the same analyses for LI versus non-LI.

For TOAST IS subtypes, we did not include AF in the second logistic regression to avoid classification bias as IS patients with AF were most likely assigned to CE subtype. If there was a significant interaction among variables, we conducted further subgroup analyses to exam risk factor associations in different groups. All the statistical hypothesis tests were two-sided, and *p* values less than 0.05 were regarded as significant. Statistical analyses were performed with R^14^.

## Results

### Characteristics of overall IS patients

We included 4953 acute IS Chinese patients into current analyses, and all had brain CT and electrocardiography (EKG). For cerebrovascular evaluation, around 60% of these patients had extracranial and intracranial CTA/MRA/angiography, while others had Carotid duplex imaging and transcranial color-coded ultrasonography. Cardiac sonography and 24-h EKG monitoring were performed in patients had cardiac disease, electrocardiography abnormality, suspected embolic stroke on brain image, sudden onset or young age of stroke, or unclear cause of stroke. The mean age was 68.1 (± 13.8) years; 59% were men. In our study, male patients had younger onset of stroke (mean age: men 66.5 ± 13.8 years, women 70.4 ± 13.6 years, *p* < 0.001), more smoking and alcohol intake, but less diabetes, AF and hyperlipidemia than female patients. The clinical characteristics and risk factor distributions were shown in Table 1.

**Table 1:** Study characteristics and risk factor distributions in ischemic stroke patients in NTUH Stroke Registry.

Mean Age (Y)	All		Male		Female		*p*-value
	68.1 (± 13.8)		66.5 (± 13.8)		70.4 (± 13.6)		*p* < 0.001
	N	(%)	N	(%)	N	(%)	
	4953		2929	59.1	2024	40.9	*p* < 0.001
Hypertension	3809	76.9% (75.7–78.7%)	2226	76.0	1583	78.2	*p* = 0.069
Diabetes	1838	37.1% (35.8–38.4%)	1052	35.9	786	38.8	*p* = 0.037
Atrial fibrillation	1180	23.8% (22.6–25.0%)	616	21.0	564	27.9	*p* < 0.001
Ischemic heart disease	759	15.3% (14.3–16.4%)	474	16.2	285	14.1	*p* = 0.044
Hyperlipidemia	1744	35.2% (33.9–36.6%)	990	33.8	754	37.3	*p* = 0.012
Smoking	1455	29.4% (28.1–30.7%)	1382	47.2	73	3.6	*p* < 0.001
Alcohol	689	13.9% (13.0–14.9%)	654	22.3	35	1.7	*p* < 0.001
Previous stroke	1175	23.7% (22.5–24.9%)	707	24.1	468	23.1	*p* = 0.409
Transient ischemic stroke	193	3.9% (3.4–4.5%)	131	4.5	62	3.1	*p* = 0.012

*NTUH* National Taiwan University Hospital, *Y* year, *N* number.

### Risk factors for the TOAST subtypes

LI was the most common subtype in the TOAST classification (27.5%), followed by CE (24.3%), undetermined (22.1%), LAA (21.0%), and other determined etiology (5.0%) (Table 2). There were significant age and sex variations among subtypes (*p* < 0.001)—CE patients were the eldest (mean 72.5 ± 13.2 years), while LAA had the highest proportion of men (67%). Different distributions were significant in all vascular risk factors studied among IS subtypes (*p* < 0.001).

**Table 2:** Study characteristics and risk factor distributions in ischemic stroke patients in the TOAST classification.

	Lacunar infarct (N = 1364)		LAA (N = 1041)		CE (N = 1203)		Other determined (N = 248)		Undetermined (N = 1097)		*p* value
	N	(%)	N	(%)	N	(%)	N	(%)	N	(%)	
Frequency	27.5%		21.0%		24.3%		5.0%		22.1%		*p* < 0.001
Mean age (Y)	67.8 (± 11.8)		70.1(± 11.4)		72.5 (± 13.2)		48.4(± 14.4)		66.2(± 14.6)		*p* < 0.001
Sex (male)	848	62.2	694	66.7	624	51.9	163	65.7	600	54.7	*p* < 0.001
Hypertension	1124	82.4	908	87.2	928	77.1	115	46.4	734	66.9	*p* < 0.001
Diabetes	556	40.8	492	47.3	379	31.5	35	14.1	376	34.3	*p* < 0.001
Atrial fibrillation	41	3.0	63	6.1	1037	86.2	10	4.0	29	2.6	*p* < 0.001
Ischemic heart disease	96	7.0	193	18.5	337	28.0	11	4.4	122	11.1	*p* < 0.001
Hyperlipidemia	634	46.5	437	42.0	280	23.3	68	27.4	325	29.6	*p* < 0.001
Smoking	434	31.8	350	33.6	270	22.4	82	33.1	319	29.1	*p* < 0.001
Alcohol	197	14.4	177	17.0	134	11.1	34	13.7	147	13.4	*p* = 0.002
Previous stroke	296	21.7	301	28.9	312	25.9	34	13.7	232	21.1	*p* < 0.001
Transient ischemic attack	43	3.2	61	5.9	53	4.4	15	6.0	21	1.9	*p* < 0.001

*Y* year, *N* number, *LAA* large artery atherosclerosis, *CE* cardioembolism.

**Figure 1:**
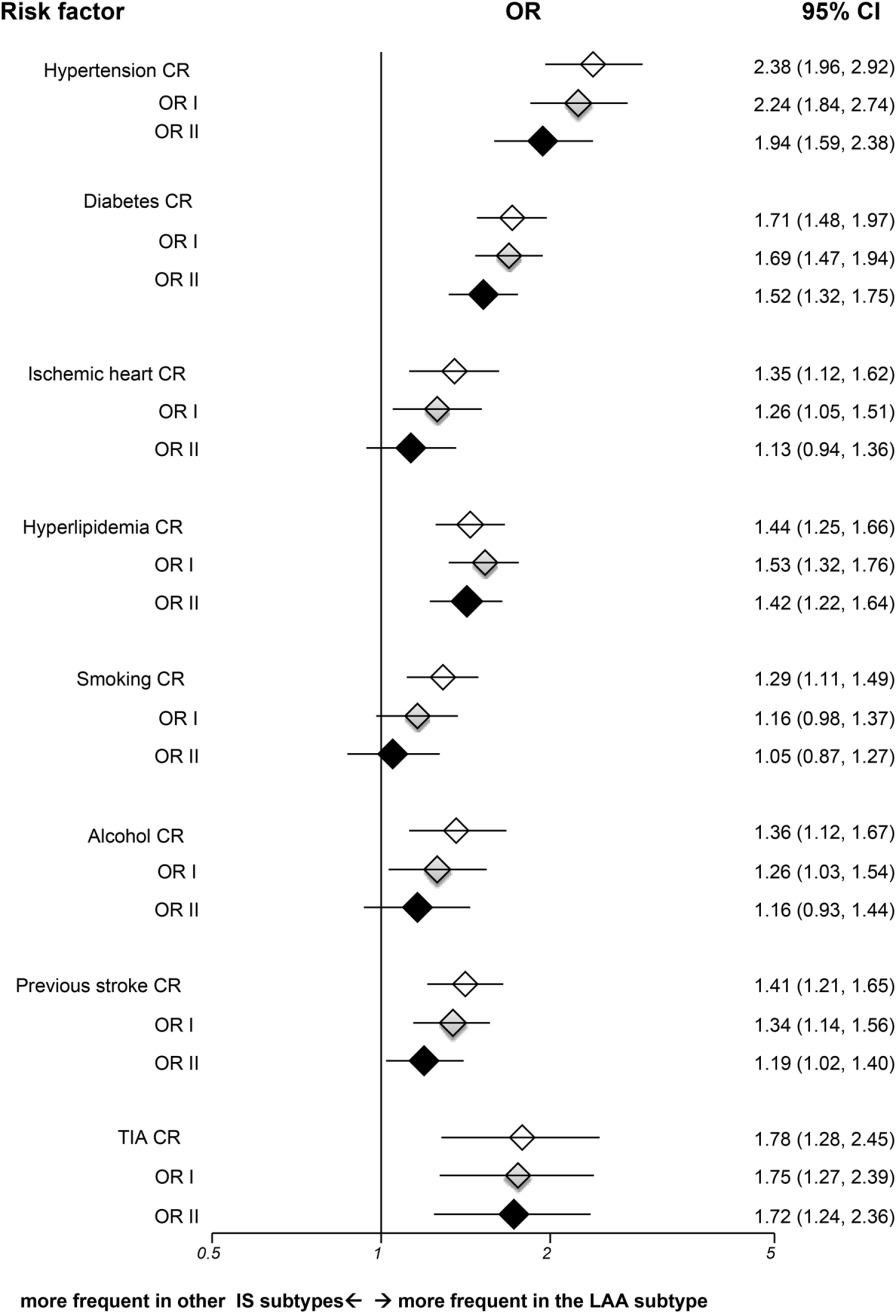
Risk factor analyses for large artery atherosclerotic strokes versus other ischemic subtypes in TOAST classification. IS = ischemic stroke; LAA = large artery atherosclerosis; OR = odds ratio; CR = crude odds ratio; OR I = adjusted odds ratio I; OR II = adjusted odds ratio II; TIA = transient ischemic attack; CI = confidence interval; Horizontal lines represent 95% CIs. Diamonds represent pooled ORs.

Compared with other IS subtypes, LAA strokes were associated with all risk factors studied (Fig. 1). After adjusting for age, sex and other risk factors, hypertension, diabetes, TIA, and hyperlipidemia had more significant associations with LAA versus others. The fully adjusted OR for hypertension was 1.94 (95% CI 1.59–2.38, *p* < 0.001), for diabetes was 1.52 (95% CI 1.32–1.75, *p* < 0.001), for TIA was 1.72 (95% CI 1.24–2.36, *p* = 0.001), and for hyperlipidemia was 1.42 (95% CI 1.22–1.64, *p* < 0.001). There was no significant interaction among variables.

**Figure 2:**
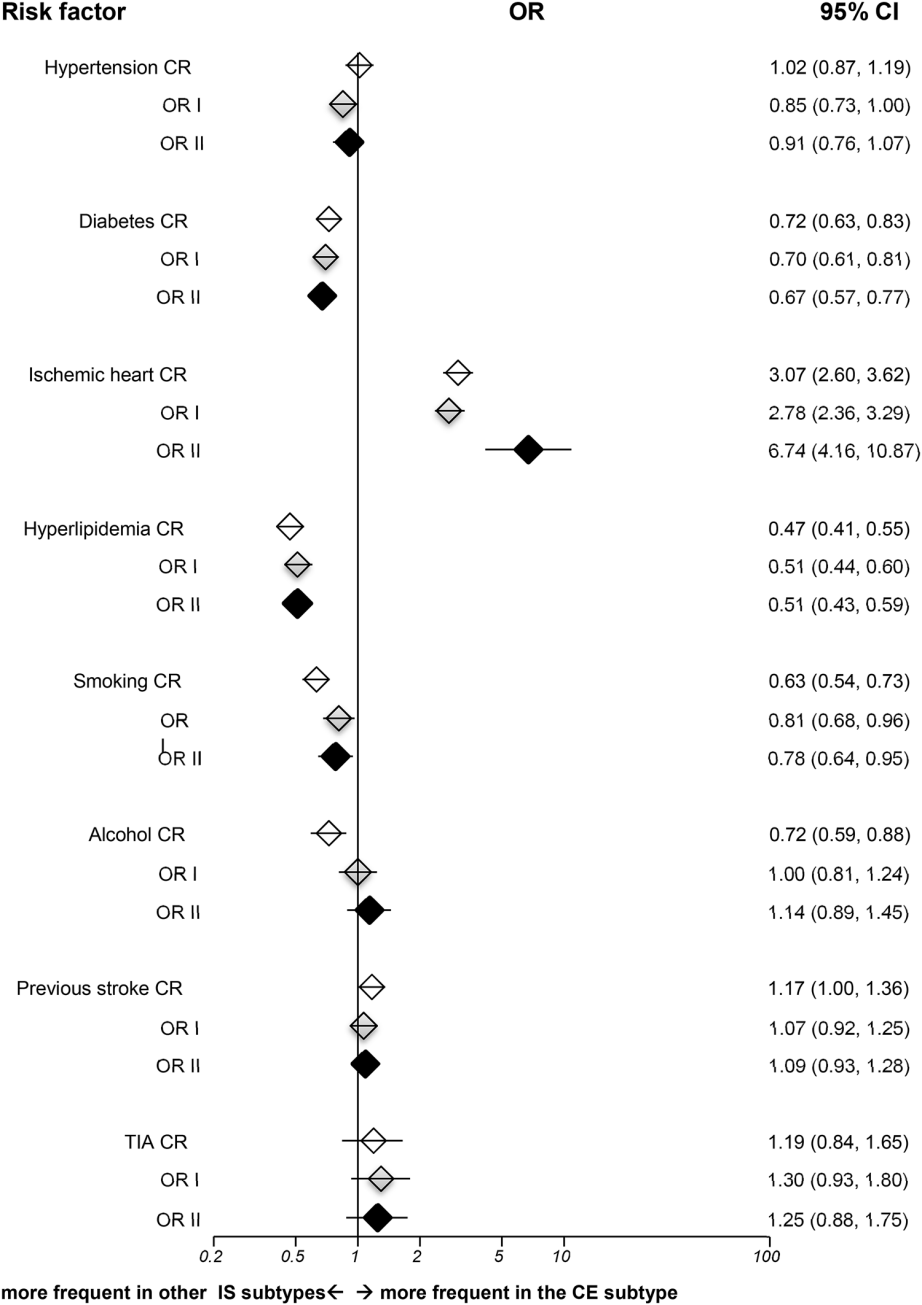
Risk factor analyses for cardioembolic strokes versus other ischemic subtypes in TOAST classification. IS = ischemic stroke; CE = cardioembolism; OR = odds ratio; CR = crude odds ratio; OR I = adjusted odds ratio I; OR II = adjusted odds ratio II; TIA = transient ischemic attack; CI = confidence interval; Horizontal lines represent 95% CIs. Diamonds represent pooled ORs.

CE strokes were more associated with age and IHD in comparison with other subtypes (Fig. 2), while having lower prevalence of male sex, diabetes, hyperlipidemia and smoking. There was a strongly significant interaction between IHD and age (*p* < 0.001). While incorporating this interaction, the fully adjusted OR for IHD was 6.74 (95% CI 4.16–10.87, *p* < 0.001).

**Figure 3:**
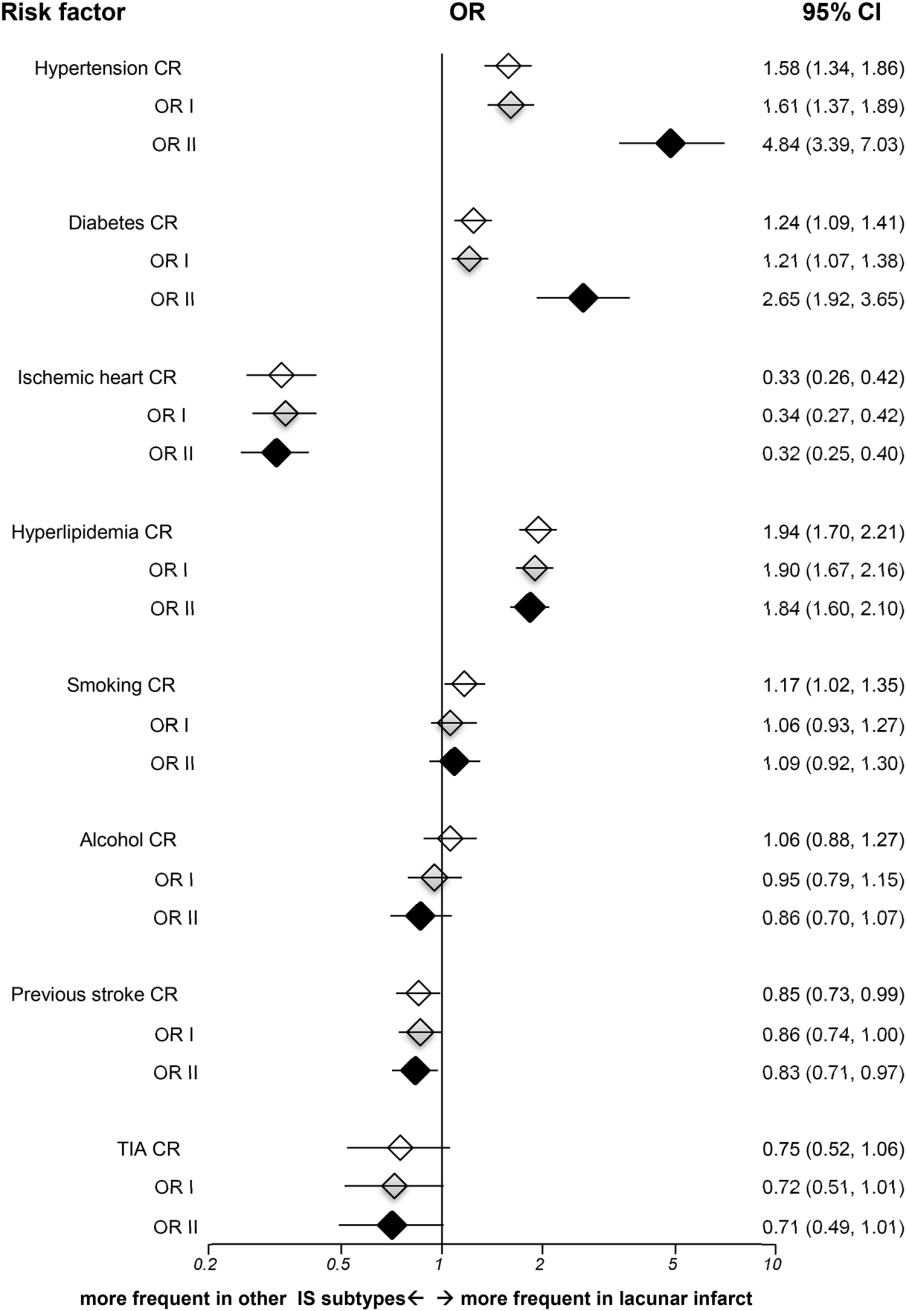
Risk factor analyses for lacunar infarct versus other ischemic subtypes in TOAST classification. IS = ischemic stroke; OR = odds ratio; CR = crude odds ratio; OR I = adjusted odds ratio I; OR II = adjusted odds ratio II; TIA = transient ischemic attack; CI = confidence interval; Horizontal lines represent 95% CIs. Diamonds represent pooled ORs.

For LI versus other IS subtypes, unadjusted analyses showed significant associations with hypertension, diabetes, and hyperlipidemia (Fig. 3), with significant interactions between age and hypertension, and between hypertension and diabetes (*p* < 0.001). While incorporating these interactions, the associations with hypertension and diabetes became stronger. The fully adjusted OR for hypertension was 4.84 (95% CI 3.39–7.03, *p* < 0.001), and for diabetes was 2.65 (95% CI 1.92–3.65, *p* = 0.025). On the contrary, the LI subtype had lower prevalence of IHD (OR II 0.32, 95% CI 0.25–0.40, *p* < 0.001).

### Risk factors for the OCSP subtypes

In the OCSP classification, ANOVA showed significant different distributions in age, male sex and all major risk factors studied in different subtypes except TIA (Supplementary Table S1). The mean age was older in TACI (69.6 ± 15.0 years), while younger in POCI (66.7 ± 13.9 years). POCI had the highest proportion of men (63%), whereas TACI possessed the lowest (51%).

**Figure 4:**
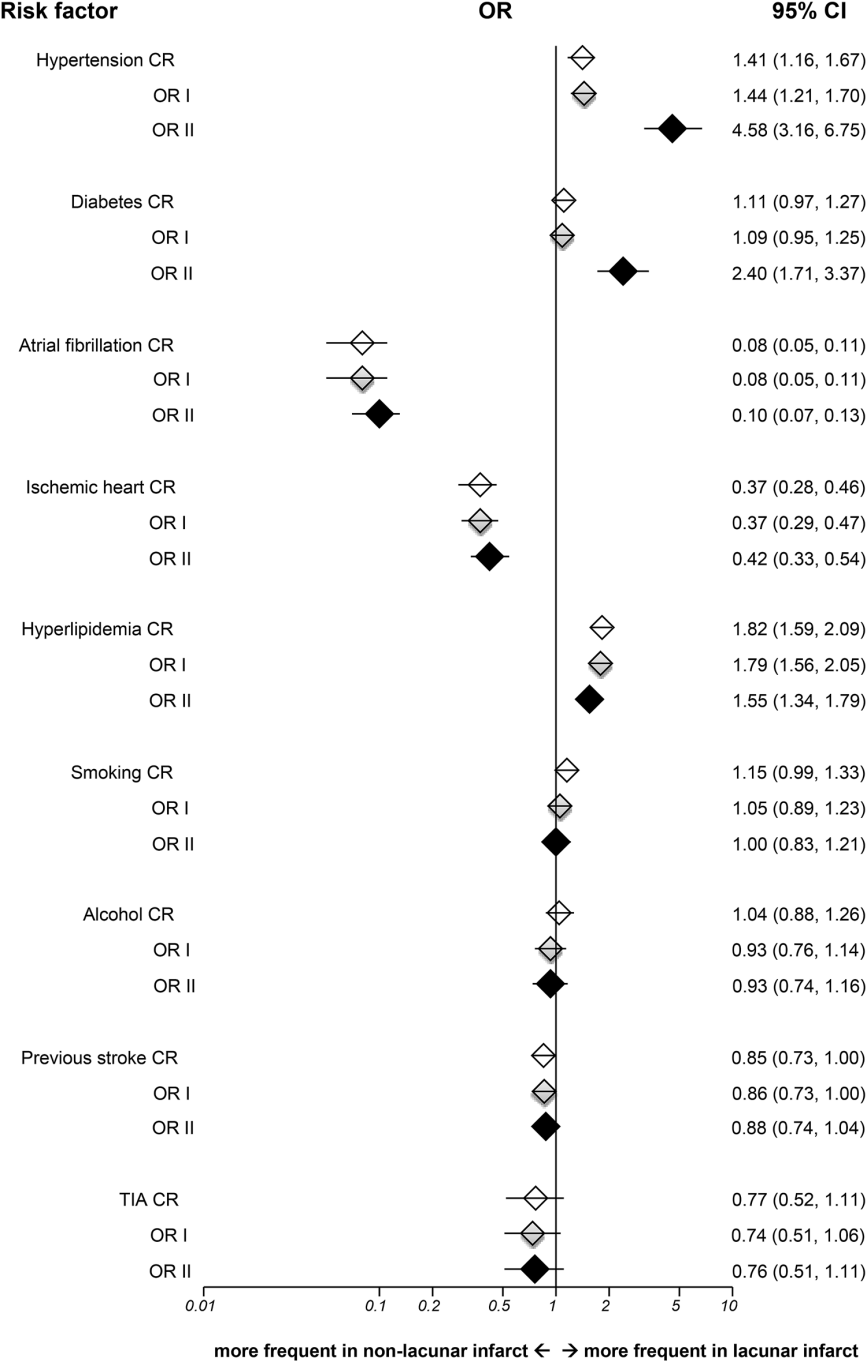
Risk factor analyses for lacunar versus non-lacunar infarct in OCSP classification. OR = odds ratio; CR = crude odds ratio; OR I = adjusted odds ratio I; OR II = adjusted odds ratio II; TIA = transient ischemic attack; CI = confidence interval; Horizontal lines represent 95% CIs. Diamonds represent pooled ORs.

In comparison to Non-LI, LI was significantly associated with hypertension (OR II 4.58, 95% CI 3.16–6.75, *p* < 0.001), diabetes (OR II 2.40, 95% CI 1.71–3.37, *p* < 0.001) and hyperlipidemia (OR II 1.55, 95% CI 1.34–1.79, *p* < 0.001), while having lower prevalence of AF (OR II 0.10, 95% CI 0.07–0.13, *p* < 0.001) and IHD (OR II 0.42, 95% CI 0.33–0.54, *p* < 0.001) in fully adjusted analysis (Fig. 4). There were significant interactions between age and hypertension, and between hypertension and diabetes (*p* < 0.001). The unadjusted and adjusted results for LI versus Non-LI in the OCSP were in line with those in the TOAST classification.

## Discussion

Our results showed that the proportions of hypertension, diabetes and hyperlipidaemia in our IS patients based in Taiwan were significantly higher, while AF, IHD, smoking and alcohol intake were lower than our previous meta-analyses for Caucasian patients^9^. As for the risk factor associations with IS subtypes, LAA and LI were more associated with hypertension, diabetes, and hyperlipidemia (varied in size), while CE strokes were more associated with IHD as compared with other subtypes. In addition, the associations with hypertension and diabetes became stronger in LI after adjusting for confounders, but not in other ischemic subtypes or in Caucasian patients. The analyses in the OCSP classification for LI versus NLI yielded consistent results.

Hypertension and diabetes were independent risk factors for LAA and lacunar strokes in our previous meta-analyses and other report^9,15^. Current study further revealed that hyperlipidemia was also a significant risk factor for both LAA and lacunar strokes in Chinese patients after adjusting for confounding factors. As compared with those in mainland China, our study showed a higher prevalence of these risk factors in IS patients based in Taiwan^9,16^. This finding was also noted in the international REACH registry—a stepwise increase of hypertension, diabetes, and hypercholesterolemia in Chinese patients moving from mainland China to Hong Kong/Singapore/Taiwan, and to North America/Western Europe^17^, which suggested that westernization of life style and diet habits probably have a heavy impact on these risk factors.

China and Taiwan may represent different stages of epidemiological transition. As infection and nutrition improve, hypertension related disease such as hemorrhagic stroke become more common in the second stage (e.g. mainland China)^18^. When the life expectancy and economics continue improving with more high-fat diet and sedentary life, atherosclerotic diseases such as diabetes and IHD increase in the third stage (e.g. Latin America)^18^. Taiwan is probably also at this stage because of earlier economic development and more influence from western countries. The epidemiological data in Taiwan could be useful to predict the disease evolution in China and other developing countries since they are experiencing rapid economic and lifestyle changes along with aging populations. As the prevalence of hypertension, diabetes, and hyperlipidemia have increased in Chinese populations in recent decades, more effective strategies through various education and intervention projects are of vital importance to reduce these risk factors and associated cerebrovascular diseases ahead^2,19–21^.

LI was the most common IS subtype in Chinese stroke patients^5^. In our analyses, there were significant interactions between age and hypertension, and between hypertension and diabetes. The associations with hypertension and diabetes became stronger for LI after incorporating the significant interactions in fully adjusted analyses in in both risk-dependent TOAST and risk-free OCSP classifications, but not in LAA or CE subtypes, or western patients^9^. These findings suggested hypertension and diabetes are strong and important risk factors for LI in Chinese patients. Our findings were different from western research, which showed no difference of hypertension and diabetes between LI and non-LI^22^, but in line with others studies in eastern Asians^23,24^. The Hisayama study in Japan also reported a greater impact of blood pressure on LI than other IS subtypes^23^. In Taiwan, the prevalence of hypertension and diabetes in IS patients had increased in the past 10 years, while the proportion of taking anti-hypertensive or anti-diabetic medication before stroke decreased^19^. Similar findings were also noted in mainland China, where only a low proportion of patients achieved the target goals of control^16^. This may be responsible (at least in part) for a higher proportion of LI in Chinese IS patients and a higher incidence of stroke in Chinese populations. Difference in genetics among ethnic groups, variations of risk factor control, and different methods of case ascertainment may account for these disparities^6–9,19,20^.

As for CE subtype, it had a totally different risk factor distribution. Our results showed CE strokes were more associated with IHD, while having lower prevalence of male sex, diabetes, hyperlipidemia, and smoking. In our analyses for TOAST IS subtypes, we did not include AF in the second logistic regression to avoid classification bias. Even though the classification system does not define subtypes explicitly based on risk factors, there is a tendency for clinicians to bias their assessments according to the strong risk factors present. For example, IS patients with AF are usually assigned to CE subtype, irrespective of other investigation results. In our study, the proportion of CE strokes was higher than previous meta-analyses for the whole Chinese patients or earlier studies, which was probably related to aging population and increasing detection of AF by echocardiography and 24-h holter monitoring^5,9,25^. The incidence of AF and related strokes are expected to increase ahead because of longer life expectancy and improved survival of IHD, but anticoagulation is still not adequate, especially in Asians^25,26^. Proper holter monitor and adequate anticoagulation may reduce the burden from CE strokes.

Our study documented various influences of risk factors on IS subtypes in Chinese populations, who had a higher incidence of stroke, a different distribution of IS subtypes, especially a higher proportion of lacunar infarct than Caucasians. Furthermore, our results showed that hypertension and diabetes were strong and important risk factors for lacunar stroke in Chinese patient. It had several strengths. First, NTUH Stroke Registry was a well-established registry, prospectively recruiting consecutive acute stroke patients with comprehensive records, without selection of age, sex or socioeconomic status as the Taiwan National Health Insurance provided affordable and compulsive medical care, covering 99% people in Taiwan. This study benefited from a relatively large number of patients, and detailed information of risk factors. Second, the inclusion of acute stroke patients was based on a standard stroke definition, having timely Neurologist and brain imaging evaluation, and in-depth investigations. Third, we used multiple logistic regressions to adjust for possible confounders, and incorporated significant interactions among variables. There were some limitations. NTUH registry was a large hospital-based stroke registry in Taiwan, which might not be fully representative of all Chinese IS patients. Also, not all acute IS patients received cardiac echography and 24-h EKG monitoring, and only few patients had prolonged EKG monitoring up to 72 h or longer. Finally, risk factors were collected from medical records before stroke, and from the included patients and their families. Whilst most patients had previous medical records, we could not totally exclude recall bias in a few patients who had no available data before stroke.

Accumulating evidence has suggested that the distributions of IS subtypes and risk factors vary among ethnicities and geographical areas, and there are different associations of risk factors with ischemic subtypes^2,5,9,15,22–24^. While genetics may play a certain role on ischemic subtypes, it is still of vital importance to target the modifiable risk factors through well-designed education and intervention projects. Herein we report various influences of risk factors on main IS subtypes in Chinese IS patients based in Taiwan. As IS accounts for the majority of heavy stroke burden in Chinese populations and around the world, our findings could help shed light on various impacts of risk factors on ischemic subtypes, reveal the potential targets to improve further, and reduce the stroke burden in the years ahead.

## Supplementary information

Supplementary Information.

## Data Availability

The datasets for this study are available from the corresponding author on reasonable request.
