# Supplementary material for: Variations of risk factors for ischemic stroke and its subtypes in Chinese patients in Taiwan
Source: Sci Rep. 2021 May 6;11:9700. doi: 10.1038/s41598-021-89228-x (PMC8102638; doi:10.1038/s41598-021-89228-x)
Supplement: Supplementary file 1 — Supplementary Information. [file 41598_2021_89228_MOESM1_ESM.docx]

**Supplementary Information**

**Variations of risk factors for ischemic stroke and its subtypes in Chinese patients in Taiwan**

Chung-Fen Tsai, MD, PhD;^a,b^ Cathie LM Sudlow, DPhil, FRCP(E);^b^ Niall Anderson, PhD;^c^ Jiann-Shing Jeng, MD, PhD^d*^

^a^ Department of Neurology, Cardinal Tien Hospital, Fu Jen Catholic University, Taiwan

^b^ Centre for Clinical Brain Sciences, University of Edinburgh, UK

^c^ Centre for Population Health Sciences, University of Edinburgh, UK

^d^ Stroke Center and Department of Neurology, National Taiwan University Hospital, Taiwan

*Corresponding Author: Professor Jiann-Shing Jeng, Stroke Center and Department of Neurology, National Taiwan University Hospital, No 7, Chung-Shan South Road, 100, Taipei, Taiwan.

Email: [chungfentsai@gmail.com](mailto:chungfentsai@gmail.com), Tel: 886-2-3123456-65338

**Supplementary Table S1. Study characteristics and risk factor distributions in ischemic stroke patients in the OCSP classification.**

|  | Lacunar infarct  (N=1162) |  | TACI  (N=1033) |  | PACI  (N=1586) |  | POCI  (N=1172) | | *p* value |
| --- | --- | --- | --- | --- | --- | --- | --- | --- | --- |
| Frequency | 23.5% | | 20.9% | | 32.0% | | 23.7% | | *p*<0.001 |
| Mean age (Y) | 67.8 (±11.8) | | 69.6 (±15.0) | | 68.3 (±14.3) | | 66.7 (±13.9) | | p<0.001 |
|  | N | (%) | N | (%) | N | (%) | N | (%) |  |
| Sex (male) | 726 | 62.5% | 524 | 50.7% | 942 | 59.4% | 737 | 62.9% | *p*<0.001 |
| Hypertension | 945 | 81.3% | 755 | 73.1% | 1173 | 74.0% | 936 | 79.9% | *p*<0.001 |
| Diabetes | 453 | 39.0% | 308 | 29.8% | 569 | 35.9% | 508 | 43.3% | *p*<0.001 |
| Atrial fibrillation | 38 | 3.3% | 549 | 53.1% | 375 | 23.6% | 218 | 18.6% | *p*<0.001 |
| Ischemic heart disease | 85 | 7.3% | 268 | 25.9% | 251 | 15.8% | 155 | 13.2% | *p*<0.001 |
| Hyperlipidemia | 535 | 46.0% | 238 | 23.0% | 502 | 31.7% | 469 | 40.0% | *p*<0.001 |
| Smoking | 367 | 31.6% | 256 | 24.8% | 480 | 30.3% | 352 | 30.0% | *p*=0.003 |
| Alcohol | 166 | 14.3% | 127 | 12.3% | 207 | 13.1% | 189 | 16.1% | *p*=0.043 |
| Previous stroke | 251 | 21.6% | 234 | 22.7% | 413 | 26.0% | 277 | 23.6% | *p*=0.041 |
| Transient ischemic attack | 37 | 3.2% | 42 | 4.1% | 69 | 4.4% | 45 | 3.8% | *p*=0.469 |

Y=year; N=number; TACI=total anterior circulatory infarct; PACI=partial anterior circulatory infarct; POCI=posterior circulatory infarct.
